# A Phase II Study of Osimertinib in Patients with Advanced-Stage Non-Small Cell Lung Cancer following Prior Epidermal Growth Factor Receptor Tyrosine Kinase Inhibitor (EGFR TKI) Therapy with EGFR and T790M Mutations Detected in Plasma Circulating Tumour DNA (PLASMA Study)

**DOI:** 10.3390/cancers15204999

**Published:** 2023-10-16

**Authors:** Yvonne L. E. Ang, Xiaotian Zhao, Thanyanan Reungwetwattana, Byoung-Chul Cho, Bin-Chi Liao, Rebecca Yeung, Herbert H. Loong, Dong-Wan Kim, James Chih-Hsin Yang, Sun Min Lim, Myung-Ju Ahn, Se-Hoon Lee, Thitiporn Suwatanapongched, Kanchaporn Kongchauy, Qiuxiang Ou, Ruoying Yu, Bee Choo Tai, Boon Cher Goh, Tony S. K. Mok, Ross A. Soo

**Affiliations:** 1 Department of Haematology-Oncology, National University Cancer Institute, Singapore 119074, Singapore; 2 Geneseeq Research Institute, Geneseeq Technology Inc., Nanjing 210032, China; 3 Division of Medical Oncology, Department of Medicine, Faculty of Medicine Ramathibodi Hospital, Mahidol University, Bangkok 10400, Thailand; 4 Division of Medical Oncology, Department of Internal Medicine, Yonsei Cancer Center, Yonsei University College of Medicine, Seoul 03722, Republic of Korea; 5 Department of Oncology, National Taiwan University Hospital, Taipei 100229, Taiwan; 6 National Taiwan University Cancer Center, Taipei 100229, Taiwan; 7 Clinical Oncology Department, Pamela Youde Nethersole Eastern Hospital, Chai Wan, Hong Kong; 8 Department of Clinical Oncology, The Chinese University of Hong Kong, Central Ave, Hong Kong; 9 Seoul National University College of Medicine, Seoul National University Hospital, Seoul 03080, Republic of Korea; 10 Division of Haematology-Oncology, Samsung Medical Center, Seoul 06351, Republic of Korea; silkahn@skku.edu (M.-J.A.); shlee119@skku.edu (S.-H.L.); 11 Division of Diagnostic Radiology, Department of Diagnostic and Therapeutic Radiology, Faculty of Medicine Ramathibodi Hospital, Mahidol University, Bangkok 10400, Thailand; 12 Clinical Research Center, Faculty of Medicine Ramathibodi Hospital, Mahidol University, Bangkok 10400, Thailand; 13 Saw Swee Hock School of Public Health, National University of Singapore, Singapore 117549, Singapore

**Keywords:** EGFR T790M mutations, Osimertinib, circulating tumour DNA, next-generation sequencing, mechanisms of resistance

## Abstract

**Simple Summary:**

Lung cancers with *EGFR* gene mutations treated with targeted therapy often develop another genetic change (T790M) that allows them to be treated with a further targeted therapy, Osimertinib, with good outcomes. The gold standard for the detection of these changes is to perform a tissue biopsy, but this is not always feasible. This study aimed to evaluate the outcomes of treatment with Osimertinib in patients who have a T790M mutation detected by non-invasive blood testing rather than tissue testing, and to explore the further genetic changes and DNA levels that can be detected in the blood during Osimertinib treatment. We demonstrated good tumour shrinkage and survival outcomes in this population, comparable to studies of patients identified through tissue testing. Levels of DNA markers in the blood before and during treatment with Osimertinib predicted outcomes. Based on this, blood testing for T790M can be used as a surrogate marker to guide Osimertinib use.

**Abstract:**

Epidermal growth factor receptor (*EGFR*) T790M mutations drive resistance in 50% of patients with advanced non-small cell lung cancer (NSCLC) who progress on first/second generation (1G/2G) EGFR tyrosine kinase inhibitors (TKIs) and are sensitive to Osimertinib. Tissue sampling is the gold-standard modality of T790M testing, but it is invasive. We evaluated the efficacy of Osimertinib in patients with EGFR mutant NSCLC and T790M in circulating tumour DNA (ctDNA). PLASMA is a prospective, open-label, multicentre single-arm Phase II study. Patients with advanced NSCLC harbouring sensitizing EGFR and T790M mutations in plasma at progression from ≥one 1G/2G TKI were treated with 80 mg of Osimertinib daily until progression. The primary endpoint was the objective response rate (ORR); the secondary endpoints included progression-free survival (PFS), overall survival (OS), disease control rate (DCR) and toxicities. Plasma next-generation sequencing was performed to determine Osimertinib resistance mechanisms and assess serial ctDNA. A total of 110 patients from eight centres in five countries were enrolled from 2017 to 2019. The median follow-up duration was 2.64 (IQR 2.44–3.12) years. The ORR was 50.9% (95% CI 41.2–60.6) and the DCR was 84.5% (95% CI 76.4–90.7). Median PFS was 7.4 (95% CI 6.0–9.3) months; median OS was 1.63 (95% CI 1.35–2.16) years. Of all of the patients, 76% had treatment-related adverse events (TRAEs), most commonly paronychia (22.7%); 11% experienced ≥ Grade 3 TRAEs. The ctDNA baseline load and dynamics were prognostic. Osimertinib is active in NSCLC harbouring sensitizing EGFR and T790M mutations in ctDNA testing post 1G/2G TKIs.

## 1. Introduction

Epidermal growth factor receptor (EGFR) tyrosine kinase inhibitors (TKIs) are the standard first-line therapy in patients with advanced non-small cell lung cancer (NSCLC) harbouring *EGFR* mutations [1]. Osimertinib, a potent mutant-specific third-generation EGFR TKI with activity towards *EGFR* sensitizing mutations and T790M mutations, is recommended as the treatment of choice in the first-line setting based on results from the FLAURA study [2]. The first- and second-generation EGFR TKIs such as gefitinib, erlotinib, afatinib, and dacomitinib remain reasonable choices, particularly in regions where Osimertinib is not readily accessible [3]. Regarding disease progression on first- and second-generation EGFR TKIs, approximately 50% of patients developed *EGFR* T790M mutations, and treatment with Osimertinib was associated with improved progression-free survival (PFS) compared with platinum-pemetrexed [4]. 

The gold standard for *EGFR* T790M testing is tissue biopsy, but this is limited by risk, feasibility, insufficient tissue sample, patient preference and the presence of tumour heterogeneity in the occurrence of resistance mechanisms [5,6,7]. Plasma testing for *EGFR* mutations offers a minimally invasive alternative to tumour testing and can be used to identify patients with T790M mutations for Osimertinib treatment [8]. A digital droplet polymerase chain reaction (ddPCR) is a quantitative method of evaluating T790M mutation status, incorporating partitioning of the PCR reaction and endpoint measurement [9]. High concordance rates are observed between ddPCR and the semi-quantitative cobas method in detecting *EGFR* mutations, with ddPCR demonstrating higher sensitivity, particularly in detecting T790M mutations in patients previously treated with EGFR TKIs [10,11].

In this study, we aimed to evaluate the efficacy of Osimertinib in patients with EGFR mutant NSCLC who have developed EGFR T790M acquired resistance to first- and second-generation *EGFR* TKIs, detected in plasma using ddPCR.

## 2. Materials and Methods

### 2.1. Trial Patients

We screened patients who were at least 21 years of age and had a histologic or cytologic diagnosis of advanced NSCLC harbouring a sensitizing *EGFR* mutation (Exon 19 deletion or Exon 21 L858R mutation) at the time of diagnosis. Patients must have had radiologic progression of disease on a prior first- or second-generation EGFR TKI treatment, ≤2 lines of prior therapy and have a sensitizing *EGFR* mutation as well as an *EGFR* T790M mutation detected in plasma using ddPCR (Sanomics) at the most recent progression. Other key inclusion criteria included adequate organ function, a life expectancy of ≥12 weeks and at least one measurable lesion based on RECIST 1.1 criteria. Patients with symptomatic brain metastases or spinal cord compression, history of interstitial lung disease, risk of QTc prolongation or other cardiac rhythm abnormalities and those who had received prior immune checkpoint inhibitors or Osimertinib were excluded. Pregnant women were excluded, and patients under study were required to have adequate contraception. All patients gave written informed consent. 

### 2.2. Trial Design, Treatment and Assessments

This was a prospective, open-label, multicentre regional single-arm Phase II study, involving eight centres in five countries and regions. Eligible patients each received Osimertinib 80 mg daily until progression (determined by RECIST v1.1), lack of clinical benefit or unacceptable toxicity. Patients underwent clinical assessments at baseline and monthly and cardiac assessments with electrocardiograms and a 2D echocardiogram or multigated acquisition scan at baseline and every 3 months. Tumour assessment by computed tomography scan was performed every 8 weeks; tumour response was assessed by investigators according to RECIST v1.1. Safety was assessed by documentation of adverse events, patient reporting, physical examination and laboratory tests. History, physical examination and blood tests for haematology and biochemistry analysis were conducted at baseline and at the start of each monthly cycle of treatment. Information on adverse events was collected from the time of consent, throughout the treatment period and until the end of the safety follow-up period, defined as 28 days after study treatment was discontinued. Adverse events were graded with the use of the Common Terminology Criteria for Adverse Events of the National Cancer Institute version 4 (NCI CTCAE v4). Plasma was taken for exploratory biomarker analysis at baseline, cycle 3 and at the end of the trial visit. 

### 2.3. Trial Endpoints

The primary endpoint of this study was the objective response rate (ORR), defined as the proportion of patients who achieved complete or partial response. The secondary endpoints included safety and tolerability and efficacy endpoints such as progression-free survival (PFS) (time from enrolment to date of documented disease progression or death from any cause), overall survival (OS) (time from enrolment to date of death from any cause), disease control rate (DCR) (proportion of patients who achieved complete response, partial response or stable disease), duration of response (DoR) (time from date of the first documented response to date of documented progression or death from any cause) and intracranial ORR. If progression or death did not occur, patients were continued in a follow-up and censored at the date of last contact or the date of study closure, whichever was earlier. This study also had the exploratory endpoints of evaluating the plasma *EGFR* dynamics and clinical outcomes and evaluating molecular alterations in serial ctDNA samples. 

### 2.4. Trial Oversight

The study was registered (NCT02811354) and approved by the independent ethics committee or institutional review board at each participating centre and was conducted in accordance with the Declaration of Helsinki and Good Clinical Practice guidelines. All patients provided written informed consent.

### 2.5. Cell-Free DNA Extract, Library Construction and Targeted Panel Next-Generation Sequencing

For whole blood samples, plasma and leukocytes were separated from other blood cells by centrifuging (at 1900× *g* for 10 min at room temperature). Cell-free DNA was extracted from 2 mL plasma using QIAamp Circulating Nucleic Acid kit (Qiagen, Germantown, MD, USA). The construction of sequencing libraries was performed using the KAPA Hyper DNA Library Prep Kit (KAPA Biosystems, Wilmington, MA, USA). Dual-indexed sequencing libraries were amplified by polymerase chain reaction (4–7 cycles), followed by purification. The size of library fragment was determined by Bioanalyzer 2100 (Agilent Technologies, Santa Clara, CA, USA). Customized probes targeting 139 cancer-relevant genes in lung cancer (Pulmocan^TM^, Nanjing Geneseeq Technology Inc., Nanjing, China) were used for hybridization enrichment. Target-enriched libraries were then sequenced on Illumina sequencing platforms (Illumina, San Diego, CA, USA) as described previously [12]. The sequencing depths of the majority of plasma samples and leukocyte samples were at least 5000× and 200×, respectively. 

### 2.6. Sequence Data Processing and Mutation Calling

FASTQ file quality control was applied with Trimmomatic [13], removing leading and trailing low-quality (quality reading <20) or N bases. High-quality reads were mapped to the reference human genome (GRCh37-hg19) using modified Burrows–Wheeler Aligner with BWA-MEM algorithm (BWA-men, v0.7.12) [14]. Deduplication was performed using Picard (v2.9.4, Broad Institute, Cambridge, MA, USA). The Genome Analysis Toolkit (GATK, v3.4.0; https://software.broadinstitute.org/gatk/ accessed on 15 May 2015) was used to locally realign the BAM files at intervals with indel mismatches and recalibrate base quality scores [15,16]. Germline mutations from leukocyte samples were identified using GATK, and somatic mutations from plasma samples were detected using VarScan2 [17]. Single-nucleotide polymorphisms were excluded when prevalence was over 1% in the 1000 Genomes Project or the Exome Aggregation Consortium (ExAC) 65,000 exomes database. Somatic variant calls with variant allele frequency (VAF) over 0.5% and at least three supporting reads were retained. When a hotspot mutation (e.g., *EGFR*, ALK, RB1, TP53) met the above threshold for somatic variant retaining in at least one sample, the threshold for the same mutation was dropped in other samples to control the false-negative rate. For plasma samples without matched germline DNA as a normal control, the mutation list was filtered by an in-house database of recurrent artifacts and common single-nucleotide polymorphisms based on approximately 500 East Asian cancer patient leukocyte sample (normal pool) sequencing values using the same target panel [18]. Annotation was performed using ANNOVAR with the hg19 reference genome [19], and each somatic mutation was checked manually with the Integrative Genomics Viewer [20]. Genomic fusions and copy number variations (CNVs) were identified using the Fusion And Chromosomal Translocation Enumeration and Recovery Algorithm (FACTERA) [21] and Aberration Detection in Tumour Exome (ADTEx) [22] with default parameters, respectively. A fold change of ≥1.6 was used to detect CNV gain, while a fold change ratio ≤ 0.6 was used to detect CNV loss.

### 2.7. Statistical Analysis

Sample size calculation was determined via the precision-based approach for the ORR. Assuming a 50% ORR with a margin of error of 10% and a 95% confidence level, a minimum sample size of 96 was required. Further accounting for a 10% dropout rate, the study would require 108 patients. In addition to intention-to-treat analysis, the ORR and DCR were analysed in patients with evaluable responses, based on modified intention to treat. Analysis for all other outcomes were on an intention-to-treat basis. The ORR, DCR and intracranial ORR were summarised in terms of frequency counts and percentage and presented with exact 95% confidence intervals (CIs) assuming binomial distribution. PFS and OS were described using the Kaplan–Meier survival curves and the estimated one-year Kaplan–Meier survival probabilities with the corresponding 95% CIs.

For exploratory analyses, odds ratios (ORs) were presented with exact 95% CIs assuming binomial distribution, and *p*-values were calculated using Fisher’s exact tests. The differences in survival across independent subgroups were described using Kaplan–Meier curves, and log-rank tests were used to compare differences. Hazard ratios (HRs) with 95% CIs were estimated using Cox proportional hazards models, and the proportionality of hazards was assessed using log(-log) survival plots. For the potential associations between genetic mutations and prognosis, multivariable Cox models controlling for patient age, sex, clinical stage and smoking history were fitted. All quoted *p*-values were two-tailed, with *p*-values < 0.05 considered to be statistically significant. Data were analysed using R software (version 4.0.3) and the *survival* and *epiR* packages.

## 3. Results

### 3.1. Patient Characteristics

**Figure A1:**
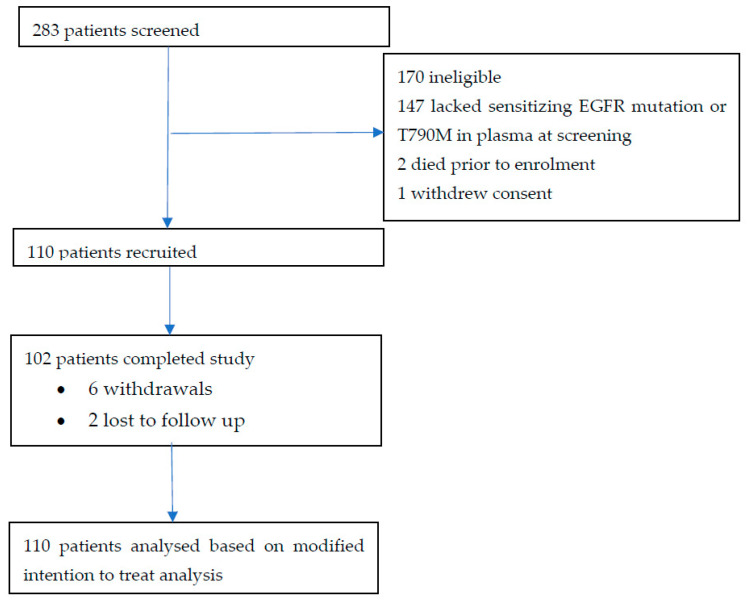
CONSORT diagram.

A total of 283 patients were screened and 110 patients were enrolled between 27 February 2017 and 7 March 2019 (Appendix A Figure A1). The median duration of follow-up was 2.6 (IQR 2.4–3.1) years, and 102 patients (92.7%) completed the study and follow-up procedures. The majority of patients were never smokers (70.9%) with ECOG performance statuses of 0–1 (90.9%), had EGFR Exon 19 deletion as the driver mutation (60.0%) and had received gefitinib previously (54.6%). Brain metastases were present in 33.3% of patients, and 27.3% of patients had received prior chemotherapy (Table 1).

### 3.2. Efficacy

**Figure 1:**
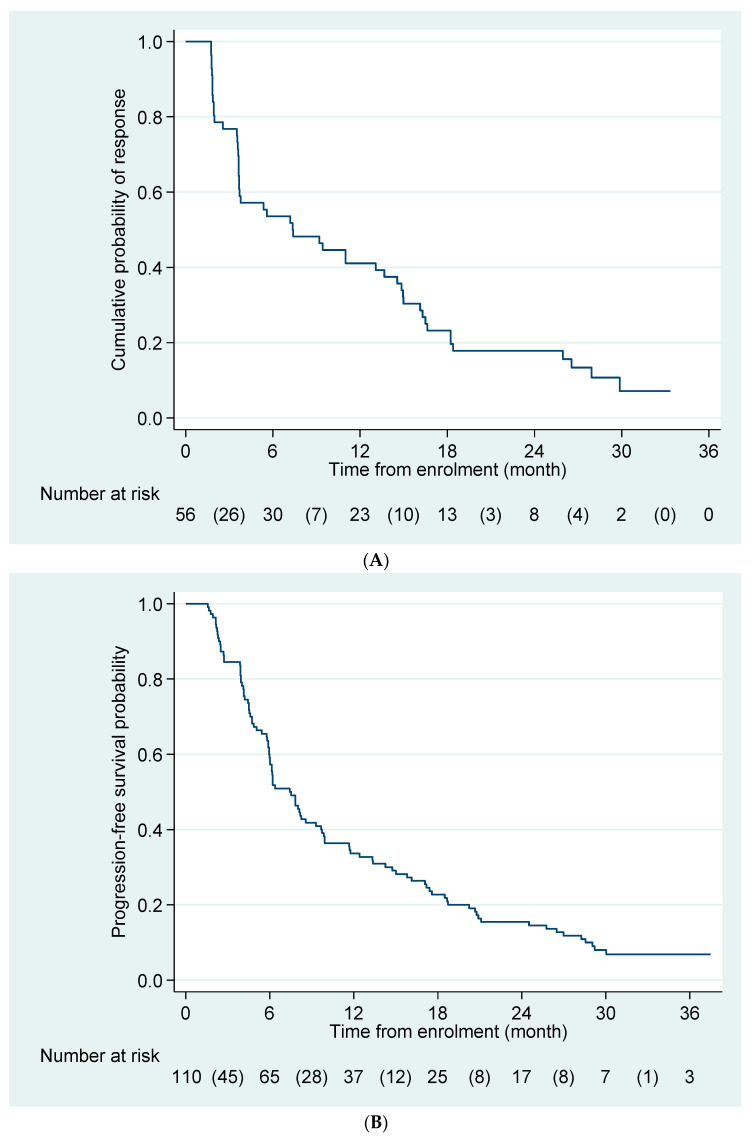
(**A**) Kaplan–Meier curve for duration of response. Shown is the Kaplan–Meier estimate of duration of response in patients who had a partial or complete response to Osimertinib (n = 56). Data for patients who had not progressed or died at the time of analysis were censored at the time of their last assessment. (**B**) Kaplan–Meier curve for progression-free survival probability. Shown is the Kaplan–Meier estimate of progression-free survival of the study population based on an intention-to-treat analysis. Data for patients who had not progressed or died at the time of analysis were censored at the time of their last assessment.

At the time of analysis, 102 of the 110 patients had progressed on Osimertinib and 77 had died; 9 patients remained on Osimertinib treatment. The ORR was 50.9% (95% CI 41.2–60.2) in the intention-to-treat (ITT) population and 61.1% (95% CI 43.5–76.9) in patients with CNS metastases. The DCR was 84.5% (95% CI 76.4–90.7) in the ITT population and 88.9% (95% CI 73.9–96.9) in patients with CNS metastases (Table 2). The median duration of response was 7.2 (95% CI 3.6–11.0) months (Figure 1A).

**Figure 2:**
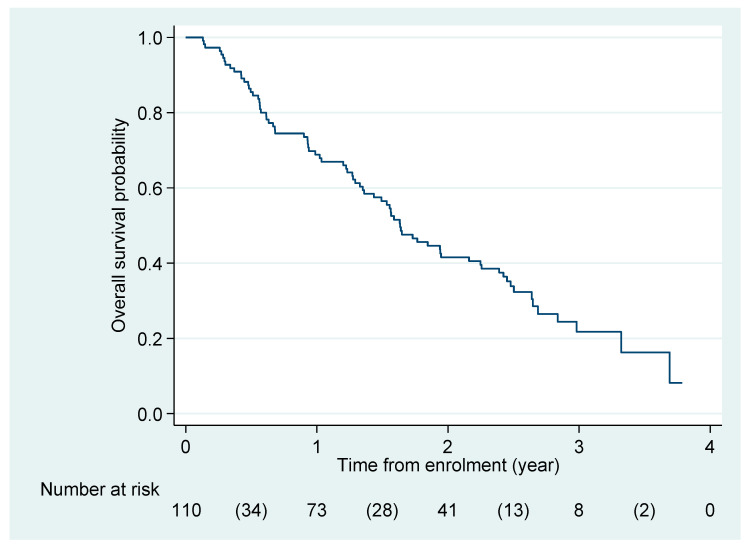
Kaplan–Meier curve for overall survival probability. Shown is the Kaplan–Meier estimate of overall survival of the study population based on an intention-to-treat analysis. Data for patients who had not died at the time of analysis were censored at the last recorded date that the patient was known to be alive.

The median PFS duration was 7.4 (95% CI 6.0–9.3) months, with a 1-year PFS probability of 33.6% (95% CI 25.0–42.5) (Figure 1B). The median OS duration was 1.63 (95% CI 1.35–2.16) years, with a 1-year OS probability of 68.8% (95% CI 59.2–76.6) (Figure 2).

### 3.3. Adverse Events and Dosing Adjustments

A total of 311 treatment-related adverse events (TRAEs) were reported in 84 patients. The toxicity profile of Osimertinib was similar to that reported in other studies, most commonly paronychia, dry skin, rash, diarrhoea and pruritis (Table 3). The TRAEs were mainly mild, with a total of 11 Grade 3 TRAEs including raised liver enzymes, reduced ejection fraction and electrocardiogram abnormalities. One patient had Grade 4 congestive cardiac failure. Osimertinib was interrupted in 14 patients (12.7%), dose reduced in 1 patient (0.9%) and discontinued in 2 patients (1.8%).

### 3.4. Exploratory Endpoints

**Figure A2:**
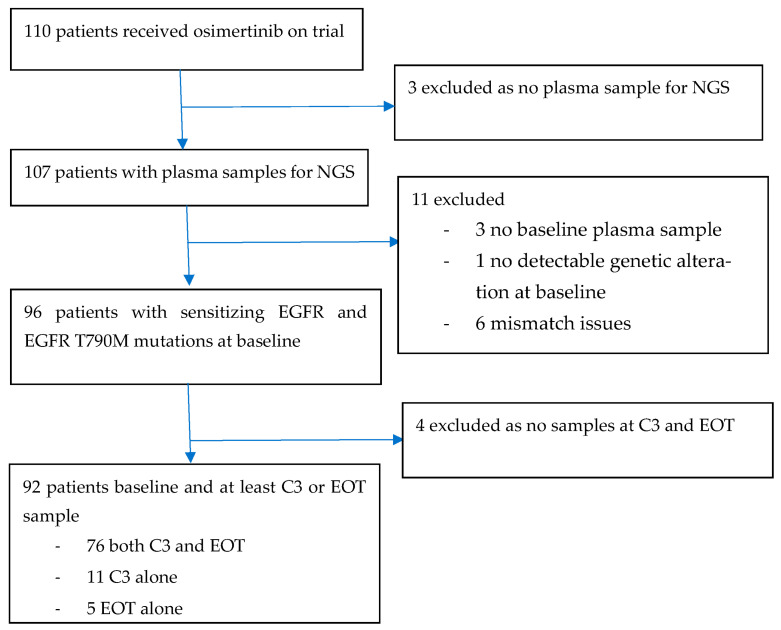
CONSORT diagram for exploratory biomarker analysis.

Plasma sampling for next-generation sequencing (NGS) and ctDNA analysis was performed. Baseline plasma NGS was performed on 107 of the 110 patients, and 96 were identified to have sensitising EGFR and T790M mutations at baseline; 92 of these were included in the serial ctDNA analysis (4 excluded as they had no follow-up sample) (Appendix A Figure A2).

**Figure 3:**
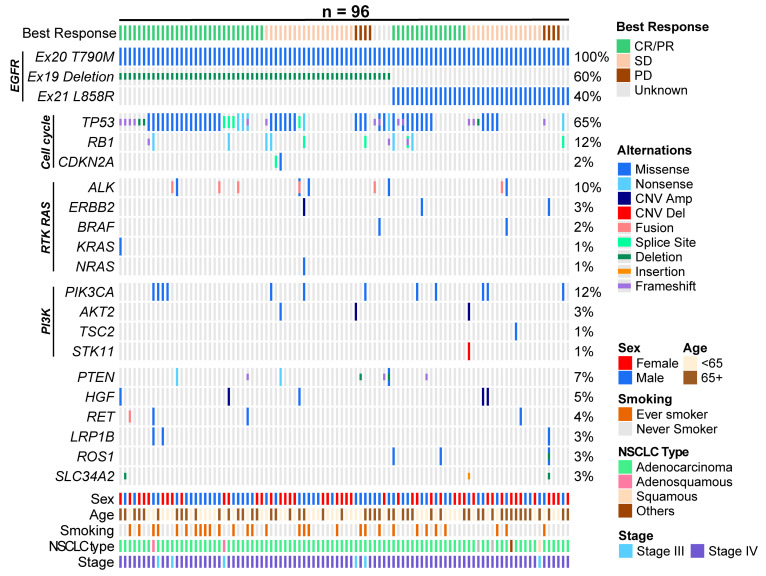
Baseline genomic characteristics profiled using NGS. This figure shows the baseline genomic and clinical characteristics for each of the 96 patients who had sensitising EGFR and T790M mutations at baseline in NGS. The best response to treatment, type of sensitising EGFR mutation, the co-occurring mutations detected on NGS and clinical characteristics (sex, age, smoking status, NSCLC type and stage) are reflected and colour-coded. Co-occurring mutations are classified into those affecting the cell cycle, the RAS pathway and the PI3K pathway.

The baseline genomics profiles of the 96 patients with sensitising EGFR and T790M mutations are shown in Figure 3. TP53 mutations were enriched in Exon 19 deletion patients vs. Exon 21 L858R mutation patients (OR 3.49, 95% CI 1.45–8.38, *p* = 0.008). Other co-occurring mutations included RB1 (12%), ALK (10%), PIK3CA (12%) and PTEN (7%). RB1, SLC34A2 and PTEN mutations at baseline were associated with poorer PFS in the multivariable analysis controlling for clinical characteristics, including patient age, sex, clinical stage at initial diagnosis and smoking history, with HRs of 2.49 (95% CI 1.25–4.98, *p* = 0.01), 5.03 (95% CI 1.39–18.13, *p* = 0.014) and 2.92 (95% CI 1.23–6.90, *p* = 0.015), respectively, while TP53 was associated with a poorer OS (HR 2.47, 95% CI 1.37–4.44, *p* = 0.003). 

**Figure 4:**
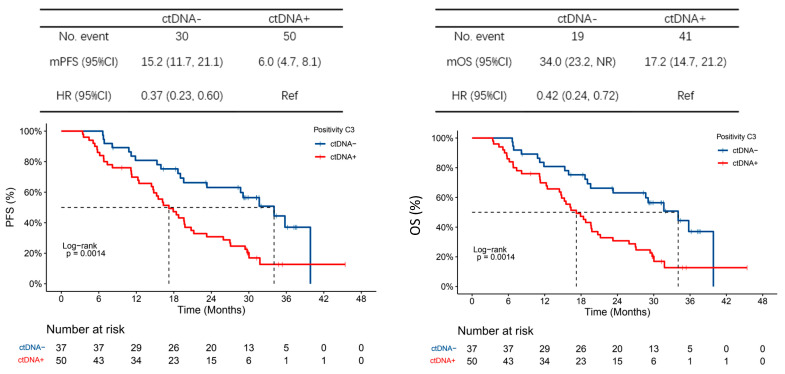
PFS and OS based on ctDNA at C3. Shown are the Kaplan–Meier estimates of progression-free survival (PFS) (**left**) and overall survival (OS) (**right**) based on whether patients were ctDNA positive (ctDNA+: blue line) or ctDNA negative (ctDNA−: red line) on plasma analysis at cycle 3. A total of 87 patients had plasma samples for analysis at cycle 3 and were included in this analysis—of these, 80 had progressed and 60 had died at the time of analysis. CI denotes confidence interval, HR denotes hazard ratio, NR denotes not reached and Ref refers to the reference population.

A higher baseline ctDNA load was associated with poorer PFS and OS. The HR for PFS of the highest quartile compared to the lowest quartile ctDNA load was 1.81 (95% CI 1.00–3.27) and the HR for OS was 2.69 (95% CI 1.31–5.52). At C3, 57.5% of patients had cleared ctDNA; 89.5% were positive for ctDNA at the end of trial (EOT). ctDNA clearance at C3 was associated with improved PFS (median 15.2 months vs. 6.0 months, HR 0.37, 95% CI 0.23–0.60) and OS (median 34.0 months vs. 17.2 months, HR 0.42, 95% CI 0.24–0.72) compared to patients who did not clear ctDNA at C3. All six patients who had not progressed at the end of the follow-up were ctDNA-negative at C3 (Figure 4). Between baseline and C3, there was a trend towards improved PFS in patients who had a decreased maximum VAF but not clearance in ctDNA compared to those who had an increase in maximum VAF (HR 0.39, 95% CI 0.15–1.00). However, OS was not significantly different (HR 1.85, 95% CI 0.57–5.88).

**Figure 5:**
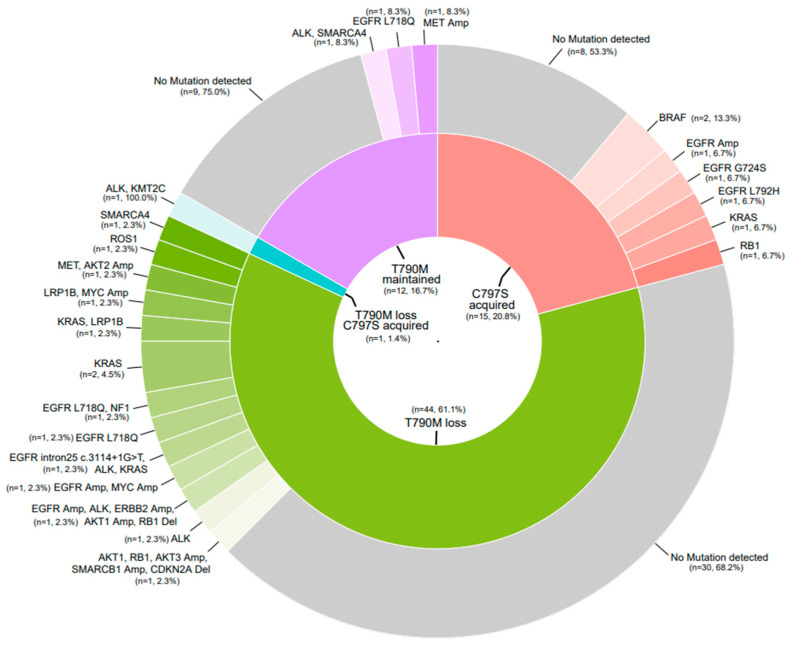
Resistance mechanisms to Osimertinib on NGS. This figure shows the mutations detected in NGS at the time of progression on Osimertinib in this study in patients who had plasma NGS performed at the end of treatment (n = 72). Patients are classified on the inner ring according to whether they had loss of T790M (n = 44), acquisition of C797S (n = 14), T790M maintained (n = 12) or T790M loss +C797S acquired (n = 1). The outer ring depicts any co-occurring mutations.

Plasma NGS at the time of Osimertinib progression was performed in 72 of the patients. Of these, 61% experienced T790M loss, 21% acquired C797S, 17% retained the T790M mutation status and one patient (1.4%) had both T790M loss and acquired C797S (Figure 5). There was a trend of longer PFS in patients with acquired C797S compared to those with loss of T790M mutations (PFS HR 0.67, 95% CI 0.38–1.20). Patients who eventually acquired C797S at progression tended to have a higher baseline T790M VAF compared to those who eventually lost their T790M status.

## 4. Discussion

This study demonstrated that Osimertinib is an effective treatment, with an ORR of 50.9% and a median PFS duration of 7.4 months in patients with sensitising *EGFR* mutations and acquired *EGFR* T790M mutations post first- or second-generation EGFR TKI, as identified by plasma ddPCR. In the AURA3 study, which identified patients based on tumour tissue biopsy, the ORR in the Osimertinib group was 71%, with a median PFS duration of 8.5 months and a median OS duration of 26.8 months [4,23]. With the limitations in cross-trial comparisons, differences may be attributed to the more heavily pretreated patients (27%) having received previous chemotherapy in this report compared to the 4% found in the AURA3 study. Our results are also consistent with a prospective Phase II study of the efficacy of Osimertinib in patients with T790M detected in *EGFR* cobas testing, regardless of tissue T790M status, which reported an ORR of 55.1% (95% CI 40.2–69.3) [8]. Multiple studies have looked at the correlation between tissue and plasma ctDNA testing for *EGFR* status. The concordance rate of *EGFR* mutations between ctDNA and tumour tissue ranges from 66% to 100%, depending on the detection technique used [24]. We used ddPCR for the detection of T790M. ddPCR has been shown to have high levels of concordance with other methods such as cobas and ARMS-PCR, with increased sensitivity in detecting T790M mutations, particularly in patients with low mutant allele frequency [10,11,25]. Thus, ddPCR has the potential to identify more patients that may benefit from Osimertinib treatment, which may otherwise have been missed by other methods. This is the first prospective study which evaluates the efficacy of Osimertinib in patients with T790M detected specifically using ddPCR. 

In a comparison between tumour and plasma *EGFR* mutation using the cobas *EGFR* mutation test and ddPCR, the sensitivity of plasma ddPCR was 81%, the specificity was 100%, the positive predictive value was 100%, and the overall concordance was 86% [26]. In a meta-analysis of 3110 patients of the diagnostic value of ctDNA vs tumour tissue, ctDNA was a highly specific and effective biomarker for the detection of *EGFR* mutation status, with a pooled sensitivity and specificity of 0.620 (95% CI 0.513–0.716) and 0.959 (95% CI 0.929–0.977), respectively [27]. 

Particularly in the context of patients with progressive disease on previous therapy, a tissue biopsy is not always feasible or may not pick up acquired resistance mutations due to tumour heterogeneity and sampling error [7]. Based on our results, and the high specificity of blood ctDNA testing, a patient who is *EGFR* positive on plasma ctDNA without accessible tissue should receive second-line Osimertinib. Sequential plasma sampling allows the study of ctDNA dynamics and repeated testing for the emergence of acquired resistance mutations in a way that tissue biopsies do not. Plasma testing also circumvents the issue of intra- and inter-tumoral heterogeneity, which can lead to false negatives due to sampling error in tissue biopsies. 

This study is limited by the increasingly common use of Osimertinib in the first line as per the FLAURA study, resulting in less opportunity to use it in the second-line setting. However, this option is still relevant, as there remain issues with the funding and accessibility of first-line Osimertinib in many countries, including the countries included in this study. First- or second-generation EGFR TKIs therefore remain reasonable first-line options, followed by second-line Osimertinib at the time of acquired resistance in patients with *EGFR* T790M mutations. In addition, out of the 110 patients enrolled, only 76 had complete sets of baseline, C3 and EOT plasma samples for analysis. This may confound our analysis of the effects of ctDNA levels on Osimertinib outcomes. This study collected only plasma samples, and plasma NGS results could not be compared with tissue. 

The strength of this study lies in the sequential collection of plasma for genomic profiling using NGS, with a relatively high proportion (69.1%) having plasma samples available for analysis at baseline, C3 and EOT. The availability of serial plasma samples allowed mapping of the evolving circulating genome with Osimertinib treatment. Concurrent *TP53*, *RB1* and *PTEN* mutations have previously been described as poor prognostic markers [28,29]. *SLC34A21* mutations have been reported previously in NSCLC and have been postulated to promote tumorigenesis via the Wnt/B catenin pathway [30]. Our study also reported on the molecular profile of the time-acquired resistance to Osimertinib in patients with *EGFR* T790M. Despite increasing the first-line use of Osimertinib, these molecular mechanisms of acquired resistance to second-line Osimertinib remain of interest and clinical importance, reflected also in the recent publication of the AURA3 NGS results [31].

We found that 21% of patients had acquired C797S, which is comparable to the 10–26% seen in other studies, including 14% in AURA3; *EGFR* T790M was undetected in 61% of cases, compared to 49% in the AURA3 population [4,23,31,32]. The rates of mutations in *KRAS* (6.9%), *BRAF* (2.8%), *ALK* (5.6%) and *ROS1* (2.8%) were similar to that reported in the literature; however, MET amplification occurred in only 2.8% of patients compared to 14% in AURA3 [31,32]. 

The exploratory analysis suggested that patients with higher T790M VAF at baseline are more likely to develop C797S resistance mutations at progression. This has not been previously reported and warrants further study, especially as newer TKI agents and strategies targeting C797S emerge. However, these findings should be interpreted with caution given the exploratory post hoc nature of the analysis and the small sample size. 

Our results show that ctDNA baseline load as well as dynamics can be prognostic, and that ctDNA clearance at C3 portends better outcomes. This is consistent with results in previous studies, which also show that molecular progression can predate and predict clinical progression [33,34,35]. The role that sequential assessment of ctDNA should play moving forward remains to be seen. Further studies looking at the escalation or de-escalation of treatment or scan intervals based on ctDNA clearance as well as the optimal thresholds and frequency of ctDNA testing are warranted. 

Interestingly, the baseline T790M load was not indicative of PFS and OS in our study. In other studies, a high baseline T790M load and high baseline *EGFR* VAF are associated with poorer outcomes, potentially due to increased tumour load [10,25,36]. This may be related to the methods of detection used. Sakai et al. found that while there were significant differences in PFS between the EGFR VAF high and low groups at baseline in ddPCR, this was not significant compared to cobas or NGS; conversely, significant differences in PFS were seen between the T790M VAF high and low groups at cycle 9 in cobas and NGS but not in ddPCR [35]. Perhaps the increased sensitivity of ddPCR affects its utility in establishing T790M VAF as a predictive marker to Osimertinib response in a way that it does not for EGFR VAF, which is present at higher levels in the plasma. The thresholds for high/low VAF also vary between studies and are not standardised. Further studies are needed to elucidate which marker—overall ctDNA load, EGFR VAF or T790M load—is the best predictor of Osimertinib response.

## 5. Conclusions

Osimertinib use guided by plasma ctDNA *EGFR* status achieved expected response rates, progression-free survival and overall survival. NGS of ctDNA enables the analysis of ctDNA dynamics and the detection of acquired resistance mutations, as ctDNA load can be prognostic.

## Figures and Tables

**Table 1:** Baseline demographic and clinical characteristics of trial participants.

Characteristics	All Patients (n = 110)
Median age (range), years	65.8 (40.7–93.7)
Gender (%)	
Male	54 (49.1)
Female	56 (50.9)
Region of participation (%)	
Hong Kong	12 (10.9)
Korea	22 (20.0)
Singapore	30 (27.3)
Taiwan	15 (13.6)
Thailand	31 (28.2)
Smoking status (%)	
Never smoker	78 (70.9)
Ex-smoker	26 (23.6)
Current smoker	6 (5.5)
ECOG performance status (%)	
0	25 (22.7)
1	75 (68.2)
2	10 (9.1)
CNS metastasis (%)	36 (33.3)
Metastasis (%)	104 (94.6)
Clinical staging at enrolment (%)	
Stage III	6 (5.5)
Stage IV	104 (94.6)
Prior therapy (%)	
Chemotherapy	30 (27.3)
Radiotherapy	54 (49.1)
Surgery	38 (34.6)
Prior TKI (%)	
Afatinib	20 (18.2)
Erlotinib	30 (27.2)
Gefitinib	60 (54.6)
EGFR mutation status (%)	
Exon 19 deletion	66 (60.0)
L858R	44 (40.0)

**Table 2:** Best tumour response.

Best Response	All Patients (n = 110)	CNS Metastasis (n = 36)
CR	1 (0.9)	0 (0)
PR	55 (50.0)	22 (61.1)
SD	37 (33.6)	10 (27.8)
PD	10 (9.1)	3 (8.3)
Not evaluable	7 (6.4)	1 (2.8)
ORR (95% CI)		
Intention to treat (ITT)	50.9 (41.2–60.2)	61.1 (43.5–76.9)
Modified intention to treat (mITT)	54.4 (44.3–64.2)	62.9 (44.9–78.5)
Median DoR (95% CI), months	7.2 (3.6–11.0)	3.6 (1.9–13.1)
DCR (95% CI)		
ITT	84.5 (76.4–90.7)	88.9 (73.9–96.9)
mITT	90.3 (82.9–95.2)	91.4 (76.9–98.2)

**Table 3:** Treatment-related adverse events occurring in ≥10% of patients.

TRAE	All Grades (n = 110)	Grade 3
Paronychia	25 (22.7%)	0
Dry skin	23 (20.9%)	0
Rash	15 (13.6%)	0
Diarrhoea	14 (12.7%)	0
Pruritis	11 (10.0%)	1 (0.9%)

## Data Availability

The data presented in this study are available on request from the corresponding author. The data are not publicly available due to maintaining the privacy of the research subjects.

## References

[1] Planchard D., Popat S., Kerr K., Novello S., Smit E.F., Faivre-Finn C., Mok T.S., Reck M., Van Schil P.E., Hellmann M.D. (2018). Metastatic non-small cell lung cancer: ESMO Clinical Practice Guidelines for diagnosis, treatment and follow-up. Ann. Oncol..

[2] Ramalingam S.S., Vansteenkiste J., Planchard D., Cho B.C., Gray J.E., Ohe Y., Zhou C., Reungwetwattana T., Cheng Y., Chewaskulyong B. (2020). Overall Survival with Osimertinib in Untreated, EGFR-Mutated Advanced NSCLC. N. Eng. J. Med..

[3] Wu Y.-L., Planchard D., Lu S., Sun H., Yamamoto N., Kim D.-W., Tan D.S.W., Yang J.C.H., Azrif M., Mitsudomi T. (2019). Pan-Aisan adapted Clinical Practice Guidelines for the management of patients with metastatic non-small-cell lung cancer: A CSCO-ESMO initiative endorsed by JSMO, KSMO, MOS, SSO and TOS. Ann. Oncol..

[4] Mok T.S., Wu Y.-L., Ahn M.-J., Garassino M.C., Kim H.R., Ramalingam S.S., Shepherd F.A., He Y., Akamatsu H., Theelen W.S.M.E. (2017). Osimertinib or Platinum-Pemetrexed in EGFR T790M-Positive Lung Cancer. N. Eng. J. Med..

[5] Chouaid C., Dujon C., Do P., MMonnet I., Madroszyk A., Le Caer H., Auliac J.B., Berard H., Thomas P., Lena H. (2014). Feasibility and clinical impact of re-biopsy in advanced non-small-cell lung cancer: A prospective multicenter study in a real-world setting (GPEC study 12-01). Lung Cancer.

[6] Chandrasekharan A., Patil V., Norohna V., Joshi A., Choughale A., Rajeev K., Mahajan A., Janu A., Goud S., More S. (2016). Rebiopsy Post Progression in EGFR Mutated Lung Cancer. J. Thorac. Oncol..

[7] Suda K., Murakami I., Obata K., Sakai K., Fujino T., Koga T., Ohara S., Hamada A., Soh J., Nishio K. (2020). Spatial heterogeneity of acquired resistance mechanisms to 1st/2nd generation EGFR tyrosine kinase inhibitors in lung cancer. Lung Cancer.

[8] Takahama T., Azuma K., Shimokawa M., Takeda M., Ishii H., Kato T., Saito H., Daga H., Tsuboguchi Y., Okamoto I. (2020). Plasma screening for the T790M mutation of EGFR and phase 2 study of osimertinib efficacy in plasma T790M-positive non-small cell lung cancer: West Japan Oncology Group 8815/LPS study. Cancer.

[9] Taylor S.C., Laperriere G., Germain H. (2017). Droplet Digital PCR versus qPCR for gene expression analysis with low abundant targets: From variable nonsense to publication quality data. Sci. Rep..

[10] Li J.Y.-C., Ho J.C.-M., Wong K.-H. (2018). T790M mutant copy number quantified via ddPCR predicts outcome after osimertinib treatment in lung cancer. Oncotarget.

[11] Thress K.S., Brant R., Carr T.H., Dearden S., Jenkins S., Brown H., Hammett T., Mireille C., Barrett J.C. (2015). EGFR mutation detection in ctDNA from NSCLC patient plamsa: A cross-platform comparison of leading technologies to support the clinical development of AZD9291. Lung Cancer.

[12] Shu Y., Wu X., Tong X., Wang X., Chang Z., Mao Y., Chen X., Sun J., Wang Z., Hong Z. (2017). Circulating tumor DNA mutation profiling by targeted next generation sequencing provides guidance for personalized treatments in multiple cancer types. Sci. Rep..

[13] Bolger A.M., Lohse M., Usadel B. (2014). Trimmomatic: A flexible trimmer for Illumina sequence data. Bioinformatics.

[14] Li H., Durbin R. (2009). Fast and accurate short read alignment with Burrows–Wheeler transform. Bioinformatics.

[15] McKenna A., Hanna M., Banks E., Sivachenko A., Cibulskis K., Kernytsky A., Garimella K., Altshuler D., Gabriel S., Daly M. (2010). The Genome Analysis Toolkit: A MapReduce framework for analyzing next-generation DNA sequencing data. Genome Res..

[16] DePristo M.A., Banks E., Poplin R., Garimella K.V., Maguire J.R., Hartl C., Philippakis A., del Angel G., Rivas M.A., Hanna M. (2011). A framework for variation discovery and genotyping using next-generation DNA sequencing data. Nat. Genet..

[17] Koboldt D.C., Zhang Q., Larson D.E., Shen D., McLellan M.D., Lin L., Miller C.A., Mardis E.R., Ding L., Wilson R.K. (2012). VarScan 2: Somatic mutation and copy number alteration discovery in cancer by exome sequencing. Genome Res..

[18] Wang H., Ou Q., Li D., Qin T., Bao H., Hou X., Wang K., Wang F., Deng Q., Liang J. (2019). Genes associated with increased brain metastasis risk in non–small cell lung cancer: Comprehensive genomic profiling of 61 resected brain metastases versus primary non–small cell lung cancer (Guangdong Association Study of Thoracic Oncology 1036). Cancer.

[19] Wang K., Li M., Hakonarson H. (2010). ANNOVAR: Functional annotation of genetic variants from high-throughput sequencing data. Nucleic Acids Res..

[20] Robinson J.T., Thorvaldsdóttir H., Winckler W., Guttman M., Lander E.S., Getz G., Mesirov J.P. (2011). Integrative genomics viewer. Nat. Biotechnol..

[21] Newman A.M., Bratman S.V., Stehr H., Lee L.J., Liu C.L., Diehn M., Alizadeh A.A. (2014). FACTERA: A practical method for the discovery of genomic rearrangements at breakpoint resolution. Bioinformatics.

[22] Amarasinghe K.C., Li J., Hunter S.M., Ryland G.L., Cowin P.A., Campbell I.G., Halgamuge S.K. (2014). Inferring copy number and genotype in tumour exome data. BMC Genom..

[23] Papadimitrakopoulou V.A., Mok T.S., Han J.-Y., Ahn M.-J., Delmonte A., Ramalingam S.S., Kim S.W., Shepherd F.A., Laskin J., He Y. (2020). Osimertinib versus platinum-pemetrexed for patients with EGFR T790M advanced NSCLC and progression on a prior EGFR-tyrosine kinase inhibitor: AURA3 overall survival analysis. Ann. Oncol..

[24] Brevet M., Johnson M.L., Azzoli C.G., Ladanyi M. (2011). Detection of EGFR mutations in plasma DNA from lung cancer patients by mass. Lung Cancer.

[25] Li Y., Xu Y., Wu X., He C., Liu Q., Wang F. (2019). Comprehensive analysis of EGFR T790M detection by ddPCR and ARMS-PCR and the effect of mutant abundance on the efficacy of osimertinib in NSCLC patients. J. Thorac. Dis..

[26] Lee C.K., Chan A.K., Park K., Leung L., Lam K.C., Yeung S.W., Lo D.Y., Mok T.S. (2013). Droplet digital PCR: A novel detection method of activating epidermal growth factor receptor (EGFR) mutations in plasma of patients with advanced stage non-small cell lung cancer (NSCLC). J. Thorac. Oncol..

[27] Qiu M., Wang J., Xu Y. (2014). Circulating Tumor DNA Is Effective for the Detection of EGFR Mutation in Non–Small Cell Lung Cancer: A Meta-analysis. Cancer Epidemiol. Biomark. Prev..

[28] Offin M., Chan J.M., Tenet M., Rizvi H.A., Shen R., Riely G.J., Rekhtman N., Daneshbod Y., Quintanal-Villalonga A., Penson A. (2019). Concurrent RB1 and TP53 alterations define a subset of EGFR-mutant lung cancers at risk for histologic transformation and inferior clinical outcomes. J. Thorac. Oncol..

[29] Chevallier M., Tsantoulis P., Addeo A., Friedlaender A. (2020). Influence of Concurrent Mutations on Overall Survival in EGFR-mutated Non-small Cell Lung Cancer. Cancer Genom. Proteom..

[30] Jiang Z., Hao Y., Ding X., Zhang Z., Liu P., Wei X., Xi J. (2016). The effects and mechanisms of SLC34A2 on tumorigenicity in human non-small cell lung cancer stem cells. Tumour Biol..

[31] Chmmielecki J., Mok T., Wu Y.-L., Han J.-Y., Ahn M.M.-J., Ramalingam S.S., John T., Okamoto I., Yang J.C.H., Shepherd F.A. (2023). Analysis of acquired resistance mechanisms to Osimertinib in patients with EGFR-mutated advanced non-small cell lung cancer from the AURA3 trial. Nat. Commun..

[32] Leonetti A., Sharma S., Minari R., Perego P., Giovannetti E., Tiseo M. (2019). Resistance mechanisms to osimertinib in EGFR-mutated non-small cell lung cancer. Br. J. Cancer.

[33] Ho H.-L., Jiang Y., Chiang C.-L., Karwowska S., Yerram R., Sharma R., Scudder S., Chiu C.-H., Tsai C.-M., Palma J.F. (2022). Efficacy of liquid biopsy for disease monitoring and early prediction of tumor progression in EGFR mutation-positive non-small cell lung cancer. PLoS ONE.

[34] Moiseenko F.V., Volkov N.M., Zhabina A.S., Stepanova M.L., Rysev N.A., Klimenko V.V., Myslik A.V., Artemieva E.V., Egorenkov V.V., Abduloeva N.H. (2022). Monitoring of the presence of EGFR-mutated DNA during EGFR-targeted therapy may assist in the prediction of treatment outcome. Cancer Treat. Res. Commun..

[35] Wu T.-H., Hsiue E.H.-C., Yang J.C.-H. (2019). Opportunities of circulating tumor DNA in lung cancer. Cancer Treat. Rev..

[36] Sakai K., Takahama T., Shimokawa M., Azuma K., Takeda M., Kato T., Daga H., Isamu O., Akamatsu H., Shunsuke T. (2021). Predicting osimertinib-treatment outcomes through EGFR mutant-fraction monitoring in the circulating tumor DNA of EGFR T790M-positive patients with non-small cell lung cancer (WJOG8815L). Mol. Oncol..

